# Transposition of Teeth

**Published:** 1853-04

**Authors:** 


*Transposition of Teeth.*—A few days since we were shown by our associate, the junior editor, a curious example of the transposition of teeth. The right superior cuspidatus occupied the place of the first bicuspis, and the last named tooth the place of the cuspidatus. The subject was a girl, a patient of his, about thirteen years of age.

